# Supplementary figures and images for: Dry season diet composition of four-horned antelope Tetracerus quadricornis in tropical dry deciduous forests, Nepal
Source: PeerJ. 2018 Jun 25;6:e5102. doi: 10.7717/peerj.5102 (PMC6022733; doi:10.7717/peerj.5102)

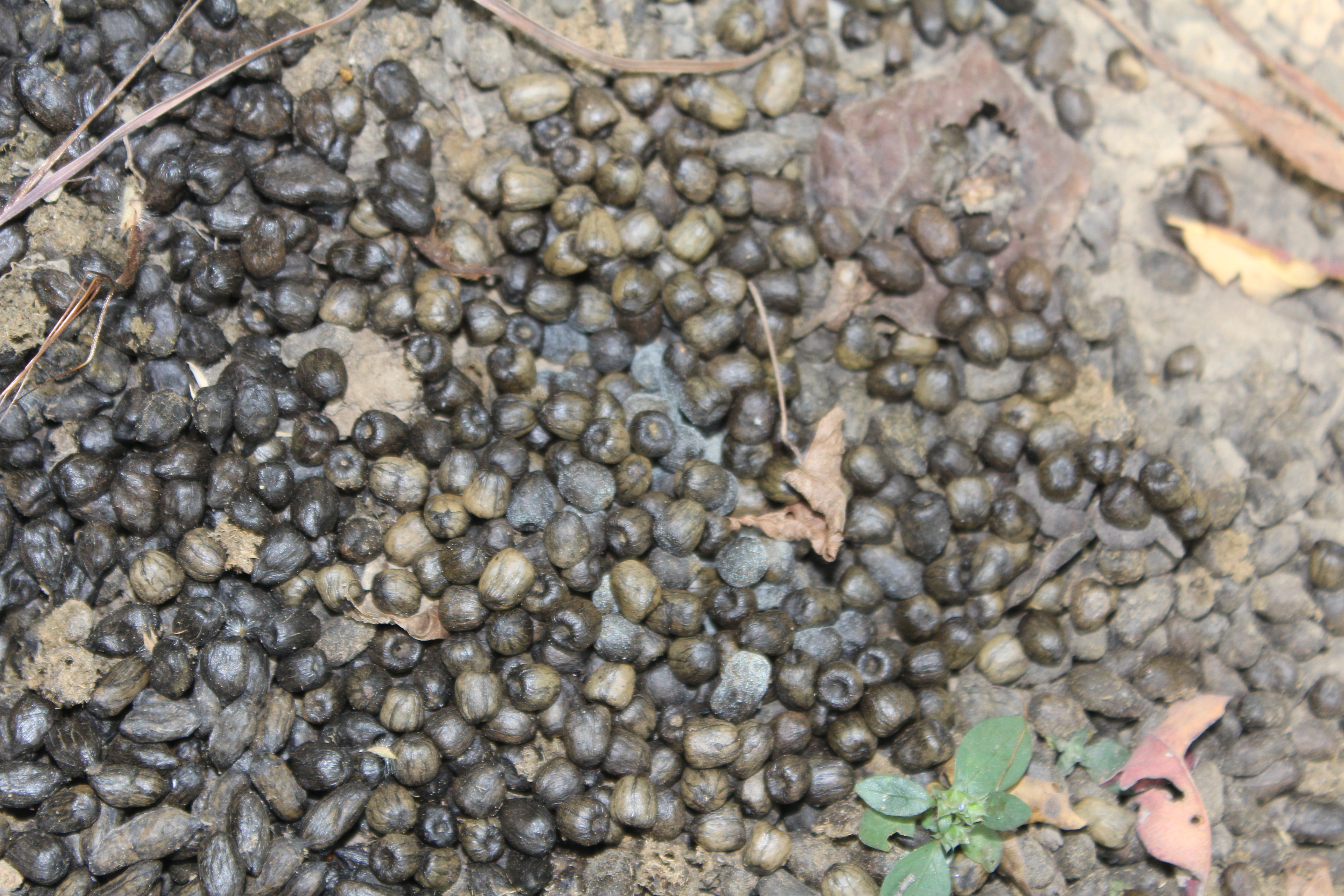

Supplement: Figure S1 [file peerj-06-5102-s002.jpg]
